# Environmental Heterogeneity as a Differential Driver for Density of Two Sympatric Rodent Species

**DOI:** 10.1002/ece3.72268

**Published:** 2025-11-11

**Authors:** Stefania Gasperini, Francesca Napoleone, Paola Bartolommei, Giorgia Bertagni, Silvia Cannucci, Linda Serafini, Sabina Burrascano

**Affiliations:** ^1^ Fondazione Ethoikos Convento dell'Osservanza snc Siena Italy; ^2^ Department of Environmental Biology Sapienza University of Rome Rome Italy; ^3^ Department of Life Sciences University of Siena Siena Italy; ^4^ NBFC National Biodiversity Future Center Palermo Italy

**Keywords:** plant functional diversity, plant functional traits, small mammals, tree‐related microhabitats, vegetation structure

## Abstract

Environmental heterogeneity drives patterns of species diversity by shifts in population abundances. High levels of heterogeneity may be associated with the disruption of ecosystem structure and, in turn, with the decline of specialist species due to a reduced availability of the habitats or resources they need. We analysed two taxonomically close and sympatric rodent species with differences in niche breadth: the forest specialist *Apodemus flavicollis* and the generalist *A. sylvaticus* . We set our study along a gradient of environmental heterogeneity across different developmental stages of a deciduous oak forest originally managed through coppicing. We assessed the densities of *Apodemus* species through spatially explicit capture‐recapture analyses in 12 grids during a three‐year campaign. We quantified environmental heterogeneity in each grid based on direct measurements of stand structural attributes, vascular plant species composition and functional traits, distinguishing between overstorey and understorey heterogeneity. We tested the responses of the species densities to these components of heterogeneity through linear mixed models. The two *Apodemus* species responded differently to environmental heterogeneity. The generalist species showed a weak decreasing trend as overstorey heterogeneity, that is, tree species diversity, increased, while the specialist species was favoured by tree diameter heterogeneity. Conversely, the highly heterogeneous understorey linked to recent coppicing was associated with higher densities of the generalist species, but understorey heterogeneity variables had no significant effect on the density of the specialist one. Our findings suggest that the response of biotic populations and communities to environmental heterogeneity may depend on the species' niche breadth and on the degree of environmental heterogeneity.

## Graphical abstract

Environmental heterogeneity acts as a differential driver for the densities of two sympatric rodent species, *Apodemus flavicollis* (specialist) and *A. sylvaticus* (generalist), along a heterogeneity gradient in a deciduous oak forest. Through a three‐year trapping campaign and analysis of forest overstorey and understorey components, we show that species responses to heterogeneity depend on niche breadth and the relative influence of understorey versus overstorey heterogeneity.

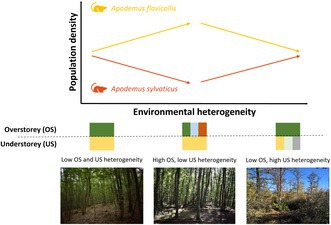

## 1 Introduction

Environmental heterogeneity has a key role in defining patterns of species diversity through complex links. For instance, the heterogeneity‐diversity hypothesis (MacArthur and MacArthur 1961) was challenged by studies incorporating population abundance data showing that over a certain level of heterogeneity, the disruption of ecosystem structure favors generalist species. On the other hand, the specialization on a particular habitat or resource limits species abundances since this habitat or resource will become scarcely available (Büchi et al. 2014), following a mechanism known as the area‐heterogeneity trade‐off (Allouche et al. 2012). While several studies tested the links between patterns of species diversity and some sources of heterogeneity, the intrinsic mechanism of decreasing species abundance as heterogeneity increases has received far less attention (Zibold et al. 2024).

Species' population abundances regulate patterns of species diversity (Supp and Ernest 2014), while they are in turn regulated by several properties of the biotic communities, for example, species niche breadth, reproduction and dispersal rates, which mediate the species responses to changes in niche dimensionality, habitat area and fragmentation (Etard and Newbold 2024; Heidrich et al. 2020; Bar‐Massada 2015).

Forests represent ideal ecosystems for the study of the effects of habitat heterogeneity on biotic communities since they include a wide range of three‐dimensional structures of different sizes that may determine a long gradient of heterogeneity as compared to other ecosystems (Zibold et al. 2024). Forest structural heterogeneity generally increases with forest age (Burrascano et al. 2017) due to a progressive accumulation of habitat structures (i.e., uneven‐aged stand structure, tree‐related microhabitats, deadwood) as forest succession proceeds towards mature and senescing phases (Norden and Appelqvist 2001). However, a major portion of forest structural heterogeneity is related to disturbance regimes (Fahey et al. 2015; Meigs and Keeton 2018), with the frequency, type and severity of disturbance playing a crucial role in driving the accumulation of complex habitat structures (Gough et al. 2022). As compared to natural disturbance, the effects of timber harvesting on forest structural heterogeneity are largely predictable since each silvicultural regime results in a specific post‐disturbance structure associated with a certain range of heterogeneity values (Chianucci et al. 2024).

Coppicing is a silvicultural approach based on stimulating the vegetative regeneration of shoots from the tree base. In some cases, standards, that is, trees of seed origin, are released to allow for a seed‐based turnover of tree individuals. Coppicing with standards has a long tradition in Europe, where it has provided rural populations with multiple resources, from wood of variable sizes obtained from shrubs and trees, to litter, and grass fodder. Such a wide range of products was linked to the high environmental heterogeneity within coppiced areas (Vollmuth 2022). Although beneficial to light‐demanding and ecotonal species (Horak et al. 2014; Chelli et al. 2021), coppiced forests are relevant for their cultural value rather than for their naturalness (Slach et al. 2021), since the spatial structure obtained through coppicing does not resemble the biological legacies usually created by forest small‐scale disturbance. Coppicing creates a fine mosaic of different habitats and ecotones expressed in the structure of the herb and shrub layers of forest vegetation, whose heterogeneity goes beyond that usually found in high forest ecosystems (Máliš et al. 2021). During the last few decades, coppiced forests largely underwent conversion to high forests, that is, promotion of regeneration through seed. The gradient of heterogeneity that could be found in historically coppiced forest landscapes is very broad and depends on both the time since the last harvesting and the type of harvesting, with the latest determining which vegetation layer contributes the most to the overall ecosystem heterogeneity. As a matter of fact, the highest overstorey heterogeneity is found in the areas converted to high forests, while understorey heterogeneity is extremely high in coppiced areas, especially after harvesting.

Here we studied the role of different components of heterogeneity in driving the population density of two sympatric rodent species: the yellow‐necked mouse *Apodemus flavicollis* and the wood mouse *A. sylvaticus* , which are morphologically and taxonomically close, but have different ecological habits that usually define them as a specialist and a generalist species, respectively. Specifically, the niche breadth of these two rodents differs for: (i) food resources, which include a more strict seed preference, especially for acorns, of *A. flavicollis* with respect to *A. sylvaticus* , which is characterised by a great foraging plasticity modifying its diet depending on the most abundant available food (Gasperini et al. 2018; Čepelka et al. 2020); (ii) habitat features, with *A. flavicollis* being more strictly associated with forested habitat with a high canopy cover as compared to *A. sylvaticus* , that can instead inhabit a wide range of different habitats including hedgerows and agricultural fields and is less sensitive to land‐use change (Marsh and Harris 2000; Sozio and Mortelliti 2016; Savazza et al. 2023).

We identified multiple parameters that could influence the abundance of the two species, ranging from food resources, that is, seed mass, to habitat structures, that is, herb and shrub vegetation used for shelter and food, lying woody debris used as natural corridors for rapid movements across the forest floor (Chambers 2002; Smith and Maguire 2004), tree‐related microhabitats used as shelters and water sources (Kraus et al. 2016; Kirsch et al. 2021). While some of these parameters may be derived from purely structural observations, others benefit from an assessment of the vegetation species and functional composition, that is, the quantification of each species cover and of those life history traits that are directly linked with ecosystem processes (Cadotte 2017). When assessing the response of animal communities to environmental heterogeneity, it is important to account for quantitative parameters of habitat conditions since the degree of heterogeneity at which a species density starts to shift depends on the quantity of the resources that the species needs, for example, habitat area, abundance of habitat structures and trophic resources (Yang et al. 2015). In this perspective, when testing the response of animal abundances, the effect of heterogeneity in structural or food resources should be analysed in the context of their overall availability (e.g., Gasperini et al. 2016; Ogawa et al. 2017). Therefore, for each parameter, we accounted for both its heterogeneity (Stevens and Tello 2011) and quantity (Clotfelter et al. 2007); for example, the effect of overstorey species diversity (heterogeneity) should be considered together with the effect of overstorey cover (quantity).

**FIGURE 1:**
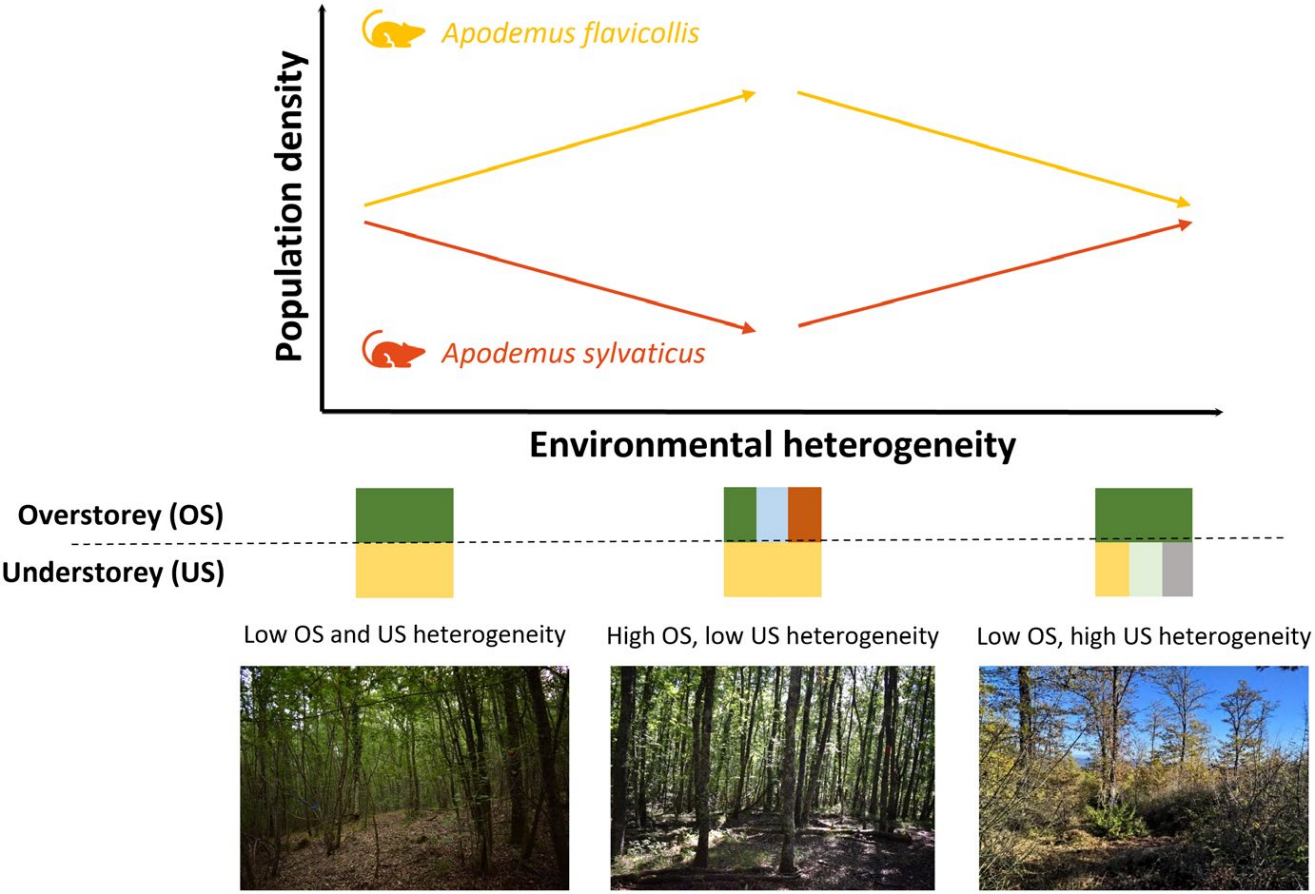
Conceptual diagram of the potential relationships between environmental heterogeneity and population density of the specialist ( *Apodemus flavicollis* ) and the generalist ( *A. sylvaticus* ) species across different sections of the environmental heterogeneity gradient dominated by overstorey and understorey heterogeneity.

Our goal is to test environmental heterogeneity as a differential driver for the density of specialist ( *A. flavicollis* ) and generalist ( *A. sylvaticus* ) species, and if this relationship varies along the heterogeneity gradient. We hypothesise that, along the broad heterogeneity gradient found in coppiced and formerly coppiced forests, the specialist species increases in density with the overstorey heterogeneity related to natural forest succession and ageing; while the generalist species increases in density with the understorey heterogeneity related to harvesting disturbance. In both cases, we expect stronger responses (i.e., higher coefficients) for the portion of heterogeneity to which each species is adapted depending on its niche breadth, that is, overstorey heterogeneity for the forest specialist *A. flavicollis* and understorey heterogeneity for the generalist *A. sylvaticus* (Figure 1).

## 2 Methods

### 2.1 Study Area

**FIGURE 2:**
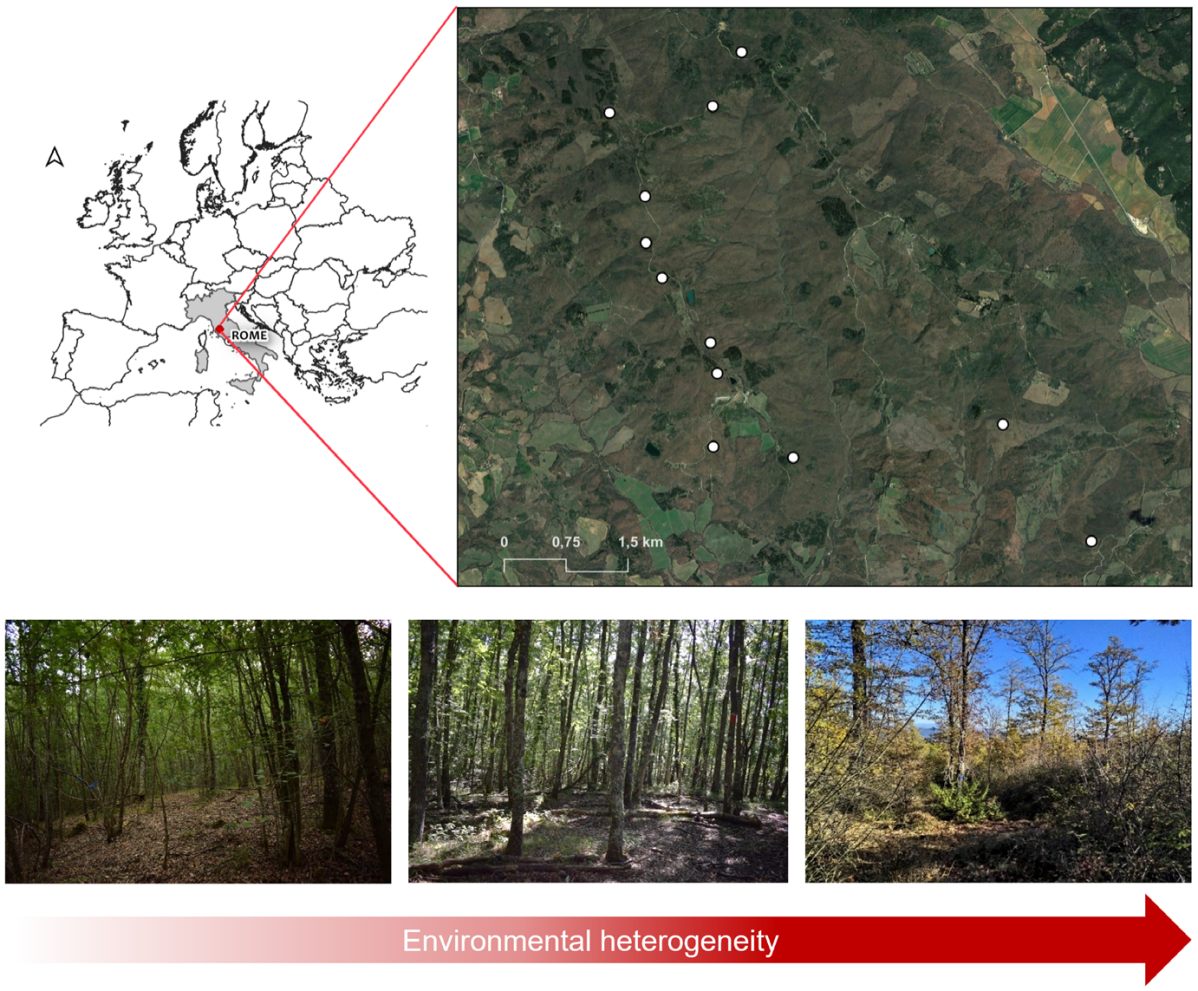
Location in Europe and satellite image of the study area. The white dots locate the position of each sampling grid within La Selva Forest (43°13′ N, 11°4′ E), 45 km west from Siena (central Italy). Bottom pictures represent, from left to right, the sampling grids distributed along an increasing gradient (red arrow) of environmental heterogeneity.

This study was conducted in a forest site of about 800 ha: La Selva Forest (43°13′ N, 11°4′ E). It is located 45 km west of Siena, in Central Italy (Figure 2), between 350 and 700 m a.s.l. The climate is Mediterranean, with warm dry summers (mean monthly temperature about 23°C) and cool wet winters (mean monthly temperature about 4°C), and an average annual rainfall of about 750–1600 mm.

The study area has long been managed as coppice with standards, with individual stands being subjected to harvesting at different points in time. Most recent interventions also include conversion to high forest and thinning. Consequently, the study site hosts a shifting mosaic of different forest regeneration phases encompassing a wide environmental heterogeneity gradient. The forest is dominated by Turkey oak ( *Quercus cerris* ), with a mixture of downy oak (*Q. pubescens*) and other deciduous tree species, that is, service tree ( *Sorbus domestica* ), wild service tree ( *S. torminalis* ), field maple ( *Acer campestre* ), field elm ( *Ulmus minor* ) and European hop hornbeam (*Ostrya carpinifolia*). According to the classification of European forest types (EEA 2006), these forests can be referred to the “downy oak forest type” (code 8.1). The understorey species composition varies across forest regeneration phases, with light‐demanding species such as *Trifolium pratense* , *Lotus corniculatus* , *L. hirsutus* and *Taraxacum vulgare* occurring in recently coppiced stands; and shade‐tolerant species such as *Ranunculus lanuginosus*, *Brachypodium sylvaticum* , *Cyclamen repandum* , *C. hederifolium* and *Helleborus viridis* occurring in mature stands. Within the forest site, we identified 12 sampling grids for rodent trapping and forest structure and vegetation sampling (Figure 2) which encompass a wide forest overstorey and understorey heterogeneity gradient deriving from the combined effect of different types of interventions (coppicing, conversion to high forest, thinning) and regeneration phases (last harvesting intervention unevenly distributed among 2 and 70 years before sampling).

### 2.2 Trapping Protocol

Trapping sessions were carried out during May, July, November and January, starting from May 2018 to July 2021 (totalling 14 sampling sessions), within 12 trapping grids, each with 49 traps (7 × 7 with traps spaced 10 m apart). During each session, traps were active for three consecutive nights. We used single‐capture live traps (Heslinga Traps) baited with a mixture of sunflower seeds, peanut butter and apple, and provided with hemp nesting material. Traps were checked daily. Captured individuals were identified at the species level and then sexed, aged, weighted and marked with a subcutaneous passive integrated transponder tag (1.25 × 8.5 mm Therapet/Bioforlife) and released at the place of capture. Molecular analyses were also performed in order to confirm the field identification of species by molecular analyses, as their visual identification is particularly challenging in southern Europe (for details, see Bartolommei et al. 2016).

The field protocol for live trapping and manipulation of animals took place in compliance with the European Council Directive 92/43EEC (Italian law DPR 357/1997 and DPR 102/2019), Italian law D.Lgs 157/92 and LR 3/1994, and was approved by Regione Toscana, with the supervision of the committee of the Italian Institute for Environmental Protection and Research (ISPRA) (Regione Toscana, DR 455/2018, DR 970/2020), and with the European Union Directive 2010/63/EU (Italian law D.Lgs 2014/26), by submission to the Animal Welfare Body of the University of Siena (Italy), and consequent permissions 396/2016‐PR and 769/2020‐PR released by the Italian Ministry of Health.

### 2.3 Vegetation and Stand Structure Sampling

We recorded: the cover of the overstorey and understorey layers, which shelter rodents from predators (Capizzi and Luiselli 1996; Gasperini et al. 2016); tree‐related microhabitats, hereafter TreMs, which are often used as a refuge and water source by rodents (Kraus et al. 2016; Kirsch et al. 2021); coarse woody debris (CWD), which is used by rodents either as shelter or as corridors to move rapidly across the forest floor (Chambers 2002; Smith and Maguire 2004).

Vegetation and stand structure sampling were performed respectively in 2021 and 2020 within two square sampling units of 400 m^2^ in each trapping grid for a total of 24 sampling units. We estimated the total cover of tree, shrub and herb layers and the cover of each individual species of vascular plants. Species not clearly identifiable in the field were dried and visually identified in the laboratory using identification keys.

Within each sampling unit we measured the Diameter at Breast Height (DBH) of all trees with a DBH > 10 cm to evaluate structural variability at the individual level, and the tree height of a sample of trees (173 across the whole site) by using a Vertex clinometer. Then, we calculated tree basal area (m^2^/ha) based on DBH measurements, as an indicator of the stand surface occupied by tree trunks, that is, the sum of the area of the trees' sections at 1.3 m height divided by stand area.

On each tree with DBH > 10 cm, we recorded the occurrence and the number of those TreMs representing a potential shelter or water source for ground‐dwelling rodents: cavities in contact with the ground, dendrotelms (cup‐shaped concavities often filled with water), root buttress cavities, distinguishing two size classes for each category (see Kraus et al. 2016).

We measured CWD length and diameter at the beginning and at the end of each fragment with the smallest diameter greater than 5 cm.

### 2.4 Understorey Vegetation Functional Trait Measurement

The vascular plant species occurring in the understorey, that is, herb, shrub and tree species growing up to a height of 3 m, build the habitat where ground‐dwelling rodents spend most of their time. Therefore, for these species, we collected data on three key functional traits (Westoby 1998): height, which indicates the structure of understorey vegetation; seed mass, which describes one of the principal foods for *A. flavicollis* and *A. sylvaticus* (Amori et al. 2008; Gasperini et al. 2018); and Specific Leaf Area (SLA), which relates to growth rate, use of light resources and successional development (Gao et al. 2022). However, based on exploratory analyses, the latter was not included in our models.

Field and laboratory measurements followed Pérez‐Harguindeguy et al. (2016). We focused on the dominant species according to the ‘mass ratio hypothesis’ (Grime 1973), and selected species with a cover equal to or greater than 2% in at least one plot. Overall, a total of 49 species were considered for the traits analysis. Plant height values were measured on 10 samples from random mature specimens of herbs and shrubs. Seed mass estimates were obtained by collecting at least 10 mature propagules (hereafter “seeds”) per species from different individuals; these were oven dried at 80°C for 48 h and subsequently weighed with a precision balance (Pérez‐Harguindeguy et al. 2016). Direct measures of seed mass were performed for 28 species, while for the other species, values were obtained from the LEDA database (https://uol.de/en/landeco/research/leda).

### 2.5 Vegetation and Stand Structure Variables

We derived values of quantity and heterogeneity of resources and habitat structure that may affect the population density of the target species (Table 1). We associated the variables related to the tree layer with forest overstorey heterogeneity and structure, while the variables related to the herb and shrub layer were identified as those related to forest understorey heterogeneity and structure. Although the latter variables can show a certain degree of variation also in closed forests (Valladares and Guzmán 2006), they are strongly related to harvesting disturbance within the study area, where different post‐harvesting regeneration phases occur (Santi et al. 2024). Quantity variables included: the total length of CWD, the total number of TreMs, tree basal area, average tree height and total cover of overstorey and understorey vegetation. For functional traits, we calculated the Community Weighted Means (CWMs) of understorey species, which represent the average trait values within the community, providing insights into the functional composition based on the dominant trait values (Garnier et al. 2004).

**TABLE 1:** Variables related to the quantity and heterogeneity of forest overstorey and understorey structure and resources.

Layer	Descriptor type	Variable	Unit	Mean	SD	Min	Max
Overstorey	Quantity	CWD total length	cm	5488.25	7426.49	360	25,418
		Number of TreM	—	9.84	8.31	1	28
		Tree basal area	m^2^/ha	6564.97	2758.4	2635.91	12,161.85
		Average tree height	m	11.16	0.48	10.33	11.84
		Overstorey cover	%	18.83	9.74	4.6	36.6
	Heterogeneity	SD CWD length	—	99.82	65.84	23.96	246.78
		Number of types of TreM	n	2.84	1.34	1	5
		SD DBH	—	6.59	2.15	3.73	11.01
		SD Tree height	m	1.44	0.46	0.7	2.19
		Overstorey Shannon index	—	2.68	1.32	1	5.48
Understorey	Quantity	Herb/Shrub CWM height	cm	258.35	81.64	156.57	465.07
		Herb/Shrub CWM seed mass	mg	144.84	66.96	59.89	287.82
		Herb/Shrub cover	%	12.07	6.19	2.95	21
	Heterogeneity	Herb/Shrub FD height	—	0.73	0.27	0.36	1.33
		Herb/Shrub FD seed mass	—	0.53	0.48	0.11	1.64
		Herb/Shrub Shannon index	—	11.84	3.51	6.42	18.44

Heterogeneity was measured using the standard deviation of CWD length, DBH and tree height, along with the number of different types of TreMs. Additionally, we calculated the Shannon index based on species composition and functional diversity for functional traits of the understorey through the packages ‘hillR’ (Li 2018) and ‘FD’ (Laliberté et al. 2014) respectively. Functional diversity was calculated as Rao's quadratic entropy and represents the diversity of functional traits across different species in the community, with implications for ecosystem stability and resilience.

### 2.6 Rodent Density Estimation

Density estimations were conducted separately for each rodent species and trapping grid. We estimated population densities (individuals/ha) with spatially explicit capture‐recapture (SECR) models (Efford 2004) that use spatial information from capture‐mark‐recapture data to estimate the distribution and density of animals in space. We ran multiple primary session models with an exponential detection function that was chosen from preliminary analysis. To facilitate statistical convergence and stability of SECR models with multiple primary sessions, we fitted models by maximizing the conditional likelihood (only groups with ≥ 5 captures during each session were included). Density was computed as a derived parameter. In trapping sessions where too few animals were captured (*n* < 5), we used the minimum number known to be alive (MNKA) divided by the average effective sampling area (ESA) estimated by the SECR models over the years (Fauteux et al. 2016). To ensure robust density estimates across grids and sessions, we adopted a parsimonious modeling approach; we thus kept both the detection function and the spatial scale parameter constant across all models, and no detection covariates were included in order to avoid overparametrization and increase model stability. The density estimations we obtained for each species, grid and session were then used as response variables in the subsequent linear mixed‐effects model analysis. All the analyses described above were performed with the R package ‘secr’ 4.5.8 (Borchers and Efford 2008; Efford 2022) in R version 4.2.2 (R Core Team 2023).

### 2.7 Statistical Analysis

We tested the responses of the densities of the two rodent species separately using as explanatory variables both quantity and heterogeneity variables in order to account for the general features of a resource when assessing species response to its heterogeneity. We used linear mixed models (LMM), taking into account the non‐independence of data by using sampling grid and sampling session as random effects.

Variables were standardized so that we could compare the betas of different variables. Overstorey and understorey variables are being treated separately for each rodent species.

For model selection, to keep our models as simple as possible we constructed sets of univariate models (individually testing all the variables), including a ‘null’ model (which did not include any slope parameter) to allow comparison of model performance to a fixed baseline (Mac Nally et al. 2018). This approach allowed for an easy interpretation of our results, given the possible complex interactions among variables as a result of collinearity (Dormann et al. 2013), and for testing the effects of variables while avoiding overparameterization (Crawley 2009). We conducted model selection using Akaike's information criterion corrected for finite samples (AICc), considering models with ΔAICc < 2 as having substantial empirical support (Anderson 2008). We also calculated Akaike weights (*w*_*i*_) for each model, to further evaluate candidate models as a measure of evidence of a particular model being the best approximating model of the set (Burnham and Anderson 2002). Top‐ranked models (< 2 ΔAICc) were model averaged to account for model selection uncertainty (Burnham and Anderson 2002). Models were built in R (R Core Team 2023) using the packages “lme4” (Bates et al. 2015) and “MuMIn” (Barton 2016).

## 3 Results

**FIGURE 3:**
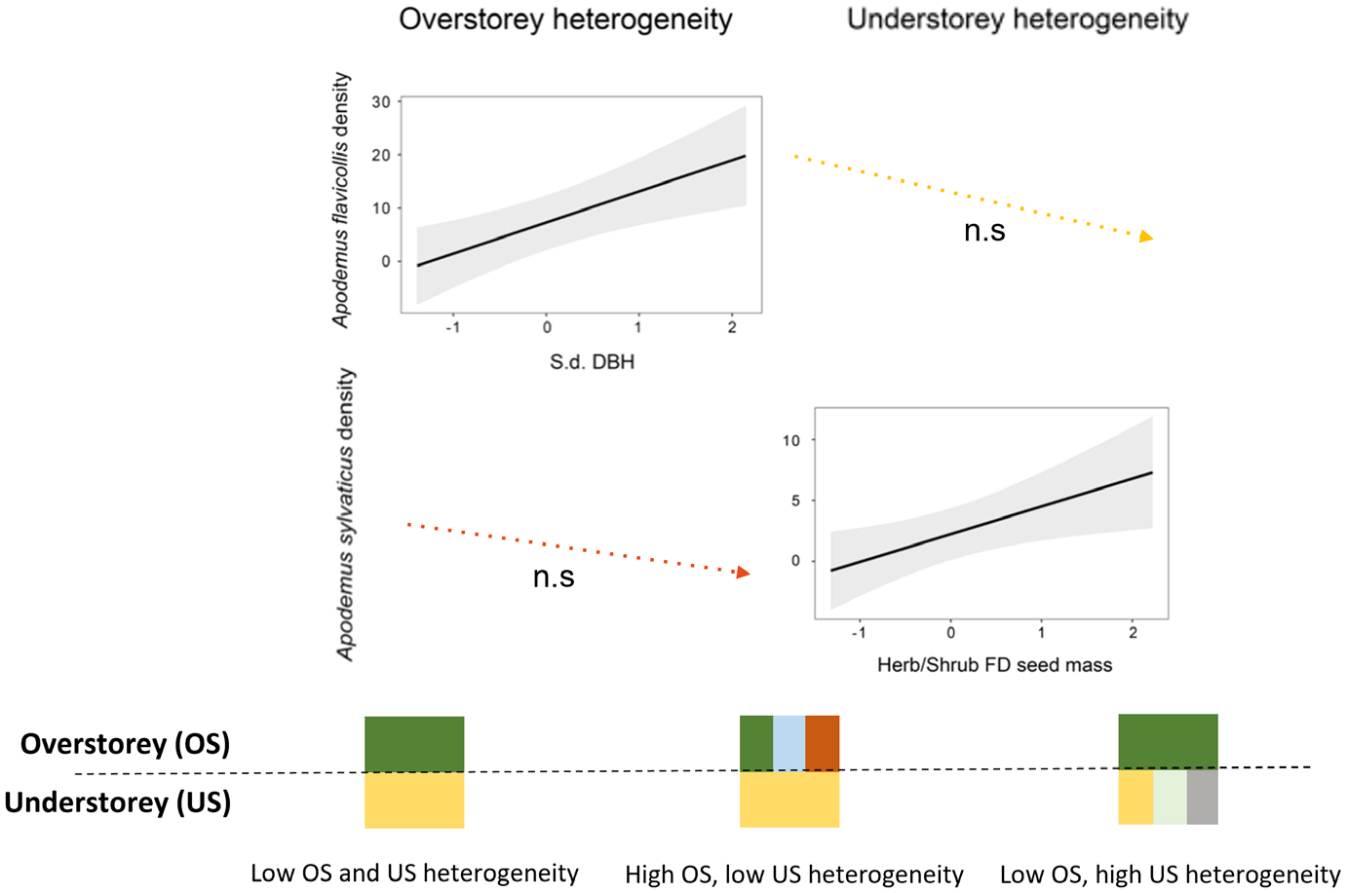
Predicted relationships between the population densities of the specialist and the generalist rodent species ( *Apodemus flavicollis* and *A. sylvaticus*, respectively) to the statistically significant overstorey and understorey variables of the top‐ranked (< 2 delta AICc, Table 2) linear mixed models, that is, tree diameter diversification for *A. flavicollis* and seed size diversification in the understorey for *A. sylvaticus* . Dotted lines represent the effect of the variables included in the top‐ranked models that resulted as not significant (n.s.), that is, understorey heterogeneity variables for *A. flavicollis* and overstorey heterogeneity variables for *A. sylvaticus* . Shading represents 95% confidence intervals.

Overall, we captured 730 individuals of *A. flavicollis* and 322 of *A. sylvaticus* over a total of 1985 capture events. Local population densities varied markedly among trapping grids, and the dynamics of both species showed intra‐ and interannual fluctuations (estimated density ind/ha: *A. flavicollis* , range 0–71; *A. sylvaticus* , range 0–49, Table S1). *Apodemus flavicollis* densities reached the highest values in relatively young and early mature stands and tended to decrease in the most mature ones and the most recently disturbed sites. On the other hand, the highest densities of *A. sylvaticus* were recorded in recently disturbed sites, while the species density decreased in closed and relatively mature sites. Rodent species showed similar intra‐ and interannual fluctuations, and both reached the highest densities in May 2019. The specialist species *A. flavicollis* responded positively to tree diameter diversification and negatively, although not significantly, to those variables expressing understorey heterogeneity, which were included in the top‐ranked models (< 2 ΔAICc) but had a higher AICc than the null one, that is, the functional diversity of understorey plant height and seed mass, and the species diversity of the understorey (Table 2, Figure 3). The generalist species, that is, *A. sylvaticus* , responded negatively to overstorey species diversity, although weakly, and positively to the diversification of seed sizes in the understorey (Table 2, Figure 3). It is important to note that while *A. sylvaticus* seems to be negatively influenced by overstorey quantitative parameters, that is, basal area, overstorey cover and total number of TreMs, *A. flavicollis* showed a weak positive response to the cover and height of the understorey layer (Table 2, Figure 3).

**TABLE 2:** Results of the LMMs relating the densities of the specialist and the generalist rodent species ( *Apodemus flavicollis* and *A. sylvaticus,* respectively) to various parameters connected to quantity and heterogeneity of forest overstorey and understorey structure and resources. Models were ranked according to AICc. Top‐ranked models (< 2 ΔAICc) and their respective AICc weight (*w*_*i*_ ) and parameter estimates are reported in bold type. “Q” refers to quantity, “H” refers to heterogeneity. “AICc” refers to Akaike's information criterion corrected for finite samples, “SE” refers to Standard Error.

	Q/H		AICc	ΔAICc	*w*_*i*_	Estimate	SE	z‐value	*p*
*Apodemus flavicollis*									
Overstorey	**H**	**SD DBH**	**1300.52**	**0**	**0.73**	**5.91**	1.87	3.16	0.01
		Null model	1305.65	5.12					
	Q	Average tree height	1306.52	6					
	H	SD CWD length	1306.6	6.08					
	Q	CWD total length	1307.25	6.73					
	Q	Tree basal area	1307.53	7.01					
	H	SD Tree height	1307.61	7.08					
	Q	Number of TreM	1307.65	7.12					
	H	Number of types of TreM	1307.74	7.21					
	H	Overstorey Shannon index	1317.76	7.23					
	Q	Overstorey cover	1317.77	7.25					
Understorey		**Null model**	**1305.65**	**0**	**0.28**				
	**H**	**Herb/Shrub FD seed mass**	**1307.08**	**1.43**	**0.14**	**−0.36**	1.29	0.28	0.78
	**Q**	**Herb/Shrub CWM seed mass**	**1307.16**	**1.51**	**0.13**	**−0.32**	1.23	0.26	0.8
	**H**	**Herb/Shrub FD height**	**1307.16**	**1.51**	**0.13**	**−0.32**	1.23	0.26	0.79
	**Q**	**Herb/Shrub cover**	**1307.34**	**1.69**	**0.12**	**0.25**	1.13	0.22	0.83
	H	Herb/Shrub Shannon index	1307.67	2.02	0.1				
	Q	Herb/Shrub CWM height	1307.75	2.1	0.1				
*Apodemus sylvaticus*									
Overstorey	**H**	**Overstorey Shannon index**	**1063.2**	**0**	**0.23**	**−0.78**	1.21	0.65	0.52
	**Q**	**Overstorey cover**	**1063.73**	**0.54**	**0.18**	**−0.57**	1.06	0.53	0.6
	**Q**	**Number of TreM**	**1064.26**	**1.06**	**0.14**	**−0.41**	0.93	0.44	0.66
	**Q**	**Tree basal area**	**1064.42**	**1.22**	**0.13**	**−0.37**	0.89	0.42	0.67
	H	Number of types of TreM	1065.51	2.32					
		Null model	1065.86	2.66					
	H	SD DBH	1065.9	2.71					
	H	SD CWD length	1066.01	2.82					
	Q	CWD total length	1067.06	3.86					
	H	SD Tree height	1067.63	4.44					
	Q	Average tree height	1067.98	4.78					
Understorey	**H**	**Herb/Shrub FD seed mass**	**1059.43**	**0**	**0.75**	**2.81**	0.8	3.54	< 0.001
	Q	Herb/Shrub CWM seed mass	1063.11	3.68					
	H	Herb/Shrub FD height	1064.49	5.06					
		Null model	1065.86	6.42					
	Q	Herb/Shrub CWM height	1066.73	7.3					
	H	Herb/Shrub Shannon index	1067.61	8.18					
	Q	Herb/Shrub cover	1067.95	8.52					

The model estimates were particularly high for the top‐ranked heterogeneity variables directly related to closed forest for *A. flavicollis* and to recently coppiced forest for *A. sylvaticus* . As a matter of fact, an estimate of 5.91 was found for the tree diameter standard deviation for the former species, while an estimate of 2.81 was found for the variability of seed size for the latter species; all the other estimates were below 0.8 (Table 2, Figure 3).

## 4 Discussion

### 4.1 The Generalist Species Responds Positively to the Heterogeneity Induced by Coppicing Disturbance

We found a positive response of the generalist species to the heterogeneity of the understorey species seed mass induced by coppicing disturbance. Woody plant seeds represent an important trophic resource for *Apodemus* species. While the forest specialist *A. flavicollis* is markedly related to acorns, *A. sylvaticus* has a greater foraging plasticity and, thanks to its broad food niche, can adapt its diet depending on the available trophic resources, e.g., herb and shrub seeds (e.g., Gasperini et al. 2016; Čepelka et al. 2020). A highly heterogeneous ecosystem structure related to timber harvesting allows for the development of both shrubs and herbs with respectively large and small seeds that may support a higher density of the feeding generalist *A. sylvaticus* (Marques et al. 2015). A greater variability of seed mass showed, instead, a weak negative relation to the density of the specialist species *A. flavicollis* ; however, it is important to note that the model including this variable, although among the top‐ranked models (ΔAICc < 2), had a slightly higher AICc than the null one. On the other hand, tree species diversity has a negative effect on the generalist species. Within the study area, the overstorey is diverse when pioneer tree species, that is, *O. carpinifolia*, *A. campestre* , *U. minor*, colonised the stand after the last harvesting intervention and persisted long enough to reach the overstorey layer. Therefore, they occur within relatively mature closed canopy stands that are not the optimal habitat for the generalist species *A. sylvaticus* (Gasperini et al. 2016), which instead thrives within the ecotones characterised by the scattered trees released during the harvesting operations, generally encompassing one or two tree species selected by the forest manager (Suchomel 2008; Tumur et al. 2007). This finding agrees with the increase of *A. sylvaticus* density in a diverse understorey with a high seed mass functional diversity (Marques et al. 2015). In accordance with our hypothesis, *A. sylvaticus* showed a weak, that is, not significant but included in the top‐ranked models, negative response to those variables whose high values indicate a closed and relatively mature forest, that is, cover of the overstorey layer, number of TreMs, tree basal area. Although not significant, these results are in accordance with studies demonstrating how a greater intensity of human disturbance influences the species response to environmental heterogeneity in relation to their niche breadth across multiple spatial scales (Maskell et al. 2023).

### 4.2 The Specialist Species Has a Positive Response to Overstorey Heterogeneity and a Negative Response to Coppicing Disturbance

The density of *A. flavicollis* was positively related to the variability of tree diameters, which generally indicates the degree of uneven agedness of the forest stands. In our study area, this variability reaches its higher values in relatively young and early mature stands, respectively coppiced approximately 13 and 25 years before this study, and tends to decrease in the most mature ones that are in the competitive exclusion phase, that is, converted to high forests more than 65 years before this study. On the other hand, the density of *A. flavicollis* decreases as the understorey heterogeneity increases due to recent copping disturbance, although models including understorey heterogeneity variables had a slightly higher AICc than the null one. As a whole, our results underline the specialisation of *A. flavicollis* to forest habitats, especially to young and early mature deciduous woodlands (Gasperini et al. 2016), suggesting a negative effect of coppicing on this species in the immediate post‐harvest phase. A meta‐analysis on the responses of small mammals to intense harvesting in Europe (Bogdziewicz and Zwolak 2014) found a slight positive effect of intense harvesting on the abundance of *A. flavicollis* in temperate forests. However, most temperate forest studies considered in the meta‐analysis were conducted in conifer‐dominated stands, while only one (Horváth et al. 2005) compared differently managed deciduous stands and found a negative response of the species to disturbance (see also, e.g., Suchomel et al. 2020; Keten et al. 2016). A meta‐analysis or review accounting for the main traits of the dominant tree species would be needed to refine the assessment of the response of *A. flavicollis* to intense harvesting.

Our results for *A. flavicollis* are generally in line with the mechanisms recalled in the area‐heterogeneity trade‐off hypothesis that assumes that, over a certain level of heterogeneity, the disruption of ecosystem structure makes the specialisation on a particular habitat or resource disadvantageous (Büchi et al. 2014). Nevertheless, the density of *A. flavicollis* shows a very weak and non‐significant decrease as understorey species and functional diversity increase. Therefore, in our study, the heterogeneity trade‐off is weak for specialist species, whose narrower niche is not likely to increase the likelihood of stochastic extinctions (Allouche et al. 2012; Ben‐Hur and Kadmon 2020).

### 4.3 Limitations and Perspectives

The variation in species density is at the base of variation in diversity patterns. In this study, we considered two rodent species that share the same habitats to explore the influence of niche breadth at driving the response of small mammals to environmental heterogeneity. Our findings could be applied to other species with caution, as we selected two model species only. A similar study, widening the focus to communities encompassing a higher number of species, would allow us to discuss in further detail the relationships between small mammal densities and the area‐heterogeneity trade‐off hypothesis.

Interestingly, the forest we selected for our study includes recently harvested stands, which may display non‐forest features that cause a decrease in structural and food resource availability for a forest specialist, while hosting dense populations of generalist species. It is interesting to note that both the closed forest stands and the recently harvested ones are universally mapped and accounted for as forests (FAO 2020) in national inventories as well as in ecological studies (Campetella et al. 2011), since they are part of a shifting mosaic of forest developmental phases driven by the periodic harvesting of different patches. In this context, it is important to acknowledge the habitat dynamic processes that may follow human or natural disturbance, since such processes will have strong effects on the temporal and spatial patterns of habitat heterogeneity and in turn on animal population densities (Agra et al. 2021).

## Author Contributions

**Stefania Gasperini:** conceptualization (lead), data curation (lead), formal analysis (lead), investigation (lead), methodology (lead), supervision (lead), writing – original draft (lead), writing – review and editing (lead). **Francesca Napoleone:** formal analysis (lead), writing – original draft (equal), writing – review and editing (equal). **Paola Bartolommei:** conceptualization (lead), investigation (lead), methodology (lead), supervision (lead), writing – original draft (equal), writing – review and editing (equal). **Giorgia Bertagni:** conceptualization (equal), data curation (lead), investigation (lead), writing – original draft (equal), writing – review and editing (equal). **Silvia Cannucci:** data curation (equal), formal analysis (equal), investigation (equal), writing – review and editing (equal). **Linda Serafini:** data curation (equal), investigation (equal), writing – original draft (equal), writing – review and editing (equal). **Sabina Burrascano:** conceptualization (lead), funding acquisition (lead), investigation (lead), methodology (lead), supervision (lead), writing – original draft (lead), writing – review and editing (lead).

## Conflicts of Interest

The authors declare no conflicts of interest.

## Supporting information


**Table S1:** Minimum and maximum estimated densities (ind./ha) of *Apodemus flavicollis* and *A. sylvaticus* for each of the 12 mouse trapping grids.

## Data Availability

The data and associated R code are available on Zenodo: https://doi.org/10.5281/zenodo.14629590.
