# Correction to “Environmental Heterogeneity as a Differential Driver for Density of Two Sympatric Rodent Species”

**DOI:** 10.1002/ece3.72606

**Published:** 2025-11-30

Gasperini, S., F. Napoleone, P. Bartolommei, G. Bertagni, S. Cannucci, L. Serafini, S. Burrascano 2025. “Environmental Heterogeneity as a Differential Driver for Density of Two Sympatric Rodent Species.” *Ecology and Evolution* 15, no. 11: e72268. 10.1002/ece3.72268.

In the Funding Statement of the published article, the CUP code was incorrect. The CUP code was listed as “J33C22001190001” but should instead read “B83C22002950007.”

This correction does not affect the results or conclusions of the paper.

We apologize for this error.

