# Supplementary material for: Environmental Heterogeneity as a Differential Driver for Density of Two Sympatric Rodent Species
Source: Ecol Evol. 2025 Nov 11;15(11):e72268. doi: 10.1002/ece3.72268 (PMC12603565; doi:10.1002/ece3.72268)
Supplement: Supplementary file 1 — Table S1: Minimum and maximum estimated densities (ind./ha) of Apodemus flavicollis and A. sylvaticus for each of the 12 mouse trapping grids. [file ECE3-15-e72268-s001.docx]

Supporting information to the paper

Gasperini S., Napoleone F., Bartolommei P., Bertagni G., Cannucci S., Serafini L. and Burrascano S. Environmental heterogeneity as a differential driver for density of two sympatric rodent species. *Ecology and Evolution*

Table S1. Minimum and maximum estimated densities (ind./ha) of *Apodemus flavicollis* and *A. sylvaticus* for each of the 12 mouse trapping grids.

|  | *Apodemus flavicollis* | *Apodemus sylvaticus* |
| --- | --- | --- |
| Grid 1 | 0.60-69.58 | 0.00-0.00 |
| Grid 2 | 0.00-0.70 | 0.47-49.07 |
| Grid 3 | 3.86-70.85 | 0.00-10.33 |
| Grid 4 | 0.00-15.56 | 0.00-0.54 |
| Grid 5 | 0.00-12.78 | 0.00-0.54 |
| Grid 6 | 0.00-10.26 | 0.00-0.54 |
| Grid 7 | 0.00-29.77 | 0.00-11.32 |
| Grid 8 | 0.00-29.20 | 0.00-0.54 |
| Grid 9 | 0.00-21.77 | 0.00-0.54 |
| Grid 10 | 0.00-49.79 | 0.00-0.41 |
| Grid 11 | 0.00-0.00 | 0.00-7.93 |
| Grid 12 | 0.00-0.70 | 0.00-43.79 |
